# Continent-Scale Sampling Reveals Fine-Scale Turnover in a Beneficial Bug Symbiont

**DOI:** 10.3389/fmicb.2020.01276

**Published:** 2020-06-19

**Authors:** Alison Ravenscraft, Margaret W. Thairu, Allison K. Hansen, Martha S. Hunter

**Affiliations:** ^1^Center for Insect Science, University of Arizona, Tucson, AZ, United States; ^2^Department of Entomology, University of California, Riverside, Riverside, CA, United States; ^3^Department of Entomology, University of Arizona, Tucson, AZ, United States

**Keywords:** symbiosis, spatial structure, *Burkholderia*, Hemiptera, *Jalysus*, environmental acquisition

## Abstract

Many members of animal-associated microbial communities, including the gut flora, are acquired from their host’s environment. While many of these communities are species rich, some true bugs (Hemiptera) in the superfamilies Lygaeoidea and Coreidae allow only ingested *Burkholderia* to colonize and reproduce in a large portion of the midgut. We studied the spatial structuring of *Burkholderia* associated with a widespread omnivorous bug genus, *Jalysus* (Berytidae). We sampled Wickham’s stilt bug, *Jalysus wickhami*, across the United States and performed limited sampling of its sister species, the spined stilt bug *Jalysus spinosus*. We asked: (1) What *Burkholderia* strains are hosted by *Jalysus* at different locations? (2) Does host insect species, host plant species, or location influence the strain these insects acquire? (3) How does *Burkholderia* affect the development and reproductive fitness of *J. wickhami*? We found: (1) Sixty-one *Burkholderia* strains were present across a sample of 352 individuals, but one strain dominated, accounting for almost half of all symbiont reads. Most strains were closely related to other hemipteran *Burkholderia* symbionts. (2) Many individuals hosted more than one strain of *Burkholderia*. (3) *J. wickhami* and *J. spinosus* did not differ in the strains they hosted. (4) Insects that fed on different plant species tended to host different *Burkholderia*, but this accounted for only 4% of the variation in strains hosted. In contrast, the location at which an insect was collected explained 27% of the variation in symbiont strains. (5) *Burkholderia* confers important fitness benefits to *J. wickhami.* In laboratory experiments, aposymbiotic (*Burkholderia*-free) insects developed more slowly and laid fewer eggs than symbiotic (*Burkholderia-*colonized) insects. (6) In the lab, nymphs sometimes acquired *Burkholderia* via indirect exposure to adults, indicating that horizontal symbiont transmission can occur via adult insect-mediated enrichment of *Burkholderia* in the local environment – a phenomenon not previously reported in bug-*Burkholderia* relationships. Taken together, the results suggest that for these bugs, critical nutritional requirements are outsourced to a highly diverse and spatially structured collection of *Burkholderia* strains acquired from the environment and, occasionally, from conspecific adults.

## Introduction

Symbioses between eukaryotes and microbes are ancient, diverse, ubiquitous, and critical to the biology of many multicellular organisms (McFall-Ngai et al., 2013; Martin et al., 2017). Examples of these symbioses are widespread within a diversity of terrestrial insects (Buchner, 1965; Douglas, 2011, 2015). Across the insect symbiosis literature, transmission mode—the way in which a host insect acquires its symbiotic microbial partner—has been emphasized as one of the major factors that determines the ecological and evolutionary costs and benefits incurred by partners in these relationships (Ebert, 2013; Salem et al., 2015). It is not, however, generally a predictor of microbial virulence (Ebert, 2013). A host insect may acquire its microbial partner vertically from its parents, horizontally from other conspecifics, or environmentally – directly from its surroundings. These different acquisition modes result in different ecological and evolutionary consequences: the evolutionary interests of a strictly vertically transmitted symbiont are likely to be closely aligned with those of its host, while a free-living microbe acquired from the environment may be pathogenic, commensal or mutualistic, depending on the microbial lineage and the host environment (Moran et al., 2008; Ebert, 2013; Salem et al., 2015).

In insects, the best-studied symbioses are stable associations maintained by vertical transmission of the symbiont from mother to offspring. However, acquisition of free-living symbionts from the environment presents a different set of potential challenges and rewards for a host (Salem et al., 2015). An insect dependent on an environmentally acquired symbiont must be able to find the microbe when the host is at the right life stage and, once the microbe is acquired, it must produce the desired benefit with few associated costs or pathogenic effects. However, the insect population might benefit from the increased flexibility of associating with many different symbiont strains that could confer different functions (Douglas, 1998). Furthermore, free-living microbes directly interact with, and adapt to, their local environment, and environmental symbiont acquisition permits a high degree of lability in partner associations. Acquisition of the right strain can therefore supply the host with instant adaptation to local conditions, and could even promote niche expansion to new diets or climates (Janson et al., 2008; White, 2011; Kikuchi et al., 2012a).

The hemipteran bug-*Burkholderia* relationship is an emerging model for the study of pairwise, environmentally acquired symbiosis (Takeshita and Kikuchi, 2017; Kaltenpoth and Flórez, 2020). Many hemipteran species in the superfamilies Lygaeoidea and Coreoidea host *Burkholderia* in sac-like outgrowths called “crypts” in the last region of the midgut, the M4 region (Kikuchi et al., 2011). Typically, after *Burkholderia* is ingested and colonizes the M4 region in the second instar nymph stage, the passage to the M4 seals and the M4 becomes a symbiotic organ rather than a flow-through gut (Ohbayashi et al., 2015; Kikuchi et al., 2020). *Burkholderia* cells are continuously pumped back into the anterior section of the gut, the M4B, where they are digested by the host as a source of nutrients (Ohbayashi et al., 2019). *Burkholderia* is a common soil inhabitant, often found in proximity to plant roots (Garcia et al., 2014) and at least the bean bug, *Riptortus pedestris*, is known to acquire *Burkholderia* from the soil (Kikuchi et al., 2007).

Strains of *Burkholderia* isolated from insects often fall into a phylogenetic clade called the stinkbug-associated beneficial and environmental (SBE) group (Kaltenpoth and Flórez, 2020). Because young nymphs acquire *Burkholderia* from the environment every generation, associations between host and symbiont species are highly labile (Kikuchi et al., 2007, 2011). While we do not know whether benefits vary among bug-*Burkholderia* systems, in the model bean bug *R. pedestris, Burkholderia* in the gut express genes associated with nitrogenous waste recycling and biosynthesis of essential amino acids and B vitamins (Ohbayashi et al., 2019). The specificity of the association with this one lineage of bacteria, the dedication of large and elaborate gut chambers (crypts) to house high densities of generally a single strain, and the high prevalence of infection (close to 100%; Kikuchi et al., 2011) of the symbiont in natural host populations suggest that *Burkholderia* provide consequential benefits in all of these associations. Rearing bugs without the symbiont has been shown to cause increased mortality and decreased size in *Riptortus* and *Alydus* (Alydidae) (Kikuchi et al., 2007; Garcia et al., 2014).

Given the high degree of lability in these relationships and their environmentally acquired nature, the factors that determine which strain a nymph will acquire are still poorly understood. We asked how the *Burkholderia* strain hosted by bugs in the genus *Jalysus* (Berytidae) varied with location, host insect species (*J. wickhami* versus *J. spinosus*), and host plant species. We characterized the alpha and beta diversity of *Jalysus*-associated *Burkholderia* strains across the United States, predicting that *Burkholderia* strains would be spatially structured as a result of climate or isolation by distance and would be correlated with the insect’s host plant species. To our knowledge, this is the largest spatial scale studied to date for a bug-*Burkholderia* symbiosis. Our results provide a better understanding of how communities of symbiotic *Burkholderia* are structured.

The *Jalysus*-*Burkholderia* symbiosis is unusual and puzzling because *Jalysus* is an omnivore, requiring both plants and scavenged or live prey for survival and reproduction (Elsey and Stinner, 1971). Many insects that depend on microbial symbionts use them to compensate for an unbalanced diet, such as a low nitrogen plant-based diet, or a B-vitamin-poor diet of blood (Buchner, 1965); however, we expect *Jalysus*’ diet to be balanced. We therefore asked whether *Burkholderia* does, in fact, provide a benefit to *Jalysus* as it has been shown to do for herbivorous bugs. We show that *Burkholderia* is indeed important for normal development and reproductive success in *Jalysus*.

## Materials and Methods

### Sample Collection and DNA Extraction

**FIGURE 1:**
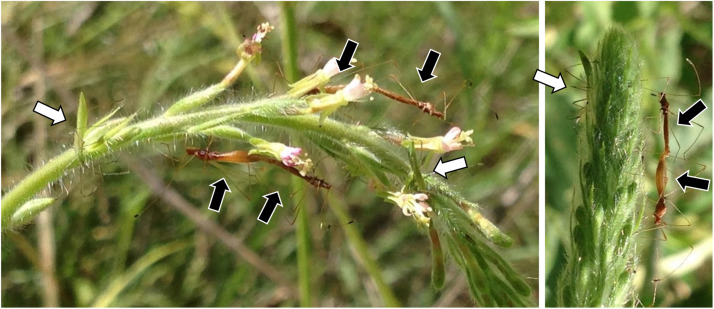
*Jalysus* in the field. Overlapping generations of *Jalysus wickhami* often live in close proximity. Here adults (black arrows) and nymphs (white arrows) are feeding on the same flower stalk of *Gaura parviflora*.

Insects were collected from 22 sites in California, Arizona, Illinois, North Carolina, or South Carolina between July 2015 and June 2017 (Supplementary Table S1) and preserved in 95% ethanol. *Jalysus* often live in aggregations with overlapping generations (Figure 1). Nymphs cannot fly and therefore usually complete development on a single host plant or patch of contiguous plants. Although adults can fly, they tend to be relatively sedentary and often return to the same host plant when disturbed. We captured 293 adults, 121 fifth instar nymphs, 43 fourth instar nymphs, and 2 third instar nymphs, plus 6 individuals for which developmental stage was not noted. This resulted in a total of 465 insects, mostly of *Jalysus wickhami* (424 individuals) but also including *Jalysus spinosus* (41 individuals).

DNA was extracted from whole insect bodies with the DNeasy Blood and Tissue Kit (Qiagen, Germantown, MD). While processing our samples, we also performed the protocol for DNA extraction on 10 empty tubes interspersed throughout the samples. These blank extractions were processed simultaneously with and identically to the samples, including all downstream lab work and sequencing, and allowed us to assess both environmental contamination and sample cross-contamination.

### Illumina Library Preparation and Sequencing

We amplified the V3-V4 hypervariable region of the 16S rRNA using the universal bacterial primer set 341f (5′-CCTACGGGNGGCWGCAG-3′) and 785r (5′-GACTACHVGGGTATCTAATCC-3′) (Klindworth et al., 2013). Primers included an overhang adapter to enable downstream addition of indexes for sample differentiation, as recommended by Illumina (Illumina Inc, 2013). Samples were amplified in a volume of 25 μL with the following reaction concentrations: 0.2 μM forward primer, 0.2 μM reverse primer, 0.2 mM dNTPs, 0.65U OneTaq Hot Start polymerase (New England Biolabs), 1X OneTaq standard reaction buffer (New England Biolabs), and 2 uL DNA extract. Thermocycler settings were: Denaturation at 95°C for 3 min followed by 30 cycles of denaturation at 95°C for 30 s, primer annealing at 55°C for 30 s, and extension at 68°C for 50 s, with a final extension of 68°C for 10 min. PCR products were visualized with gel electrophoresis and cleaned with magnetic beads (Rohland and Reich, 2012). In addition to the extraction blanks, every 96-well plate of samples included at least one blank sample of PCR water. All blanks were included in all downstream lab work and sequencing.

In a second short amplification, each sample was indexed with a unique pair of 8-nt barcodes attached to the forward and reverse strands (Hamady et al., 2008). We amplified 2.5 uL of each cleaned PCR product in a volume of 25 μL with 0.2 μM of a barcoded forward primer, 0.2 μM of a barcoded reverse primer, 0.2 mM dNTPs, 0.65U OneTaq Hot Start polymerase, and 1X OneTaq standard reaction buffer. The thermocycler program was the same as that of the initial amplification, with two modifications: the annealing temperature was 50°C and 8 amplification cycles were performed. PCR products were again visualized with gel electrophoresis and cleaned with magnetic beads.

The cleaned, dual-indexed 16S rRNA amplicons were quantified fluorometrically (Qubit DS DNA HS assay, Qiagen) and an equal mass of each sample was bidirectionally sequenced on an Illumina Mi-Seq platform using 2 × 300 chemistry, with samples split between two runs: one at the University of Arizona Genetics Core and one at the Arizona State Genomics and Bioinformatics Core.

### Sequence Data Processing

We used the program *cutadapt* (Martin, 2011) to remove priming sites and poor quality bases at the 5′ and 3′ ends of the sequences. The resulting sequences were processed in R using the “DADA2” package (Callahan et al., 2016) as follows: Using the *filterAndTrim* function, reads were truncated at the first instance of a quality score less than or equal to 2, and reads that contained any unassigned bases (Ns) or had an overall expected error score higher than 2 were discarded. We used the package’s eponymous DADA2 algorithm which employs the run-specific error rate, quality scores, and the number of times each sequence was observed to infer the true biological sequences that were present, allowing analysis at the level of bacterial strains (Callahan et al., 2016). Analysis of sequence variants, rather than use of older clustering methods that generate taxonomic units at a 97 or 99% similarity cutoff, is the current recommended best practice for amplicon data (Knight et al., 2018). Forward and reverse reads were merged and sequences less than 401 base pairs in length were discarded, resulting in a median sequence length of 427 base pairs. *De novo* chimera checking and removal were performed with the *removeBimeraDenovo* function. Taxonomy was assigned using the RDP classifier with the SILVA nr v123 database as the training set (Wang et al., 2007; Quast et al., 2013).

Eleven *Burkholderia* sequence variants were detected in the extraction and PCR blanks (Supplementary Figure S1). These included some of the most common sequence variants in the dataset, including SV1. The most common bacteria present in a library of samples are almost always detected in Illumina blanks due to phenomena such as index-swapping (Costello et al., 2018) and low-levels of sample cross-contamination. However, the sequence variants varied markedly across blanks: ten out of eleven were only detected in a single blank. SV1, the most common sequence variant in the dataset, was detected in 5 out of 15 blanks. To identify true contaminants, we compared the prevalence of each sequence variant in insect samples versus blanks using the R package “decontam” with default parameters (Davis et al., 2018). Eighteen sequence variants, including one *Burkholderia* (SV156), were classified as contaminants. These were removed from the dataset.

### Molecular Phylogenetic Analysis

We constructed a phylogeny of all *Burkholderia* sequence variants that were present in the dataset after rarefaction to 800 reads along with selected representatives of different *Burkholderia* from NCBI’s GenBank (taken from Kuechler et al., 2016). Accession numbers are available in Supplementary Table S2. Sequences of the 16S rRNA gene were aligned using MAFFT (Katoh and Standley, 2013) with the default settings. Maximum-likelihood phylogenetic trees were inferred with the GTR+Gamma model of nucleotide substitution using RAxML version 8.2.10 (Stamatakis, 2014) on the CIPRES Science Gateway (Miller et al., 2010). Node support values were calculated using rapid bootstrapping, which was halted automatically based on the MRE criterion.

### Insect Rearing Experiments

*Jalysus wickhami* proved difficult to maintain in an experimental setting and mortality of young nymphs was high (Supplementary Table S3). (However, mortality did not differ between the symbiotic and aposymbiotic treatments; Fisher’s exact test, odds ratio = 1.26, *p* = 0.6). We therefore performed three iterations of an experiment to compare the development time and/or reproductive output of *J. wickhami* reared with and without symbiotic *Burkholderia*. Rearing conditions are summarized in Table 1. In the first two experiments, a single first-instar nymph – freshly hatched from an egg in an aseptic container – was placed in a clean plastic box that contained a tomato leaflet in water and a source of animal protein (Supplementary Figure S2).

**TABLE 1:** Experimental rearing conditions.

Condition	Iteration 1	Iteration 2	Iteration 3
Temperature	28°C	Ambient (∼22°C)	28°C
Light:Dark (hours)	16:8	Ambient	16:8
Relative Humidity	65%	Ambient	65%
Protein source	Dead *Drosophila*	Artificial diet (beef)	Dead *Drosophila*
*Burkholderia* source	Single adult male (instars 1–3)	Live cells mixed into diet (instars 1–3)	Leaves exposed to six adults (instars 1–3)
Direct contact with adults?	Yes	No	No
Life stage at start	1st instar	1st instar	egg
Nymphs reared per box	1	1	Up to 8 until fifth instar, then 1

In the first iteration, dead *Drosophila* flies were provided as the protein source. From the first through the third instars, each nymph assigned to the “symbiont positive” treatment was reared in the same box as a single adult male which was normally infected with *Burkholderia*. These adult males were collected from our laboratory colony of *Jalysus wickhami*, in which insects freely acquired *Burkholderia* from the potting soil in which the host plant (tomato) was grown. Each male was maintained on a single tomato leaf – which had not previously been exposed to *Jalysus* – in the plastic rearing box for several days prior to introduction of the experimental nymph. Males that died were replaced with another male from the lab colony. In contrast, nymphs assigned to the aposymbiotic (“symbiont negative”) treatment were kept on leaves which were not exposed to any adult insects. This experiment was performed in an incubator kept at 28°C with a 16:8 hour day:night cycle and 65% relative humidity. Upon reaching adulthood, insects were immediately preserved in 95% ethanol for later DNA extraction to determine *Burkholderia* infection status.

In the second iteration of the experiment, an artificial diet of homogenized calf liver, fatty beef, and sugar water was provided as the protein source (Cohen, 1985). From the first through the third instars, a suspension of live *Burkholderia* cells was mixed into the diet of nymphs in the symbiont positive treatment; an equal volume of distilled water was mixed into the diet of the aposymbiotic nymphs. Symbiotic insects were infected with *Burkholderia* strain TF1N1, which we isolated from insects collected on tobacco in North Carolina. This strain was 100% identical in sequence (across 408 bp of the V3-V4 region of the 16s rRNA) to SV1 in our Illumina amplicon data. The rearing experiment was performed in the laboratory at room temperature (∼22°C) under ambient light and humidity conditions. Upon reaching adulthood, males were preserved in 95% ethanol for later DNA extraction. Females were maintained for an additional 2 weeks to collect data on reproductive output. A normally infected male from the lab colony was added to the cage of each female and the insects were allowed to mate freely. Eggs were collected and counted every other day. After 2 weeks, the females were preserved in 95% ethanol.

Since only six females (out of an initial 48 nymphs) successfully completed the reproductive output measurement in the second iteration, we performed a third iteration to obtain more data on reproductive fitness. To provide a source of the symbiont, six adult *J. wickhami* were maintained on a tomato leaf in a plastic box for a minimum of 5 days; these adults (and any eggs they had laid) were then removed from the box and eight fresh, experimental eggs were placed in the box. For the aposymbiotic treatment, leaf sprigs were kept in a box without adults for a minimum of 5 days prior to egg introduction. Nymphs in both treatments were switched to a new box (that was either exposed or un-exposed to six adults, as appropriate) every three to four days until the end of the third instar. At the beginning of the fifth instar, each nymph was transferred to a fresh box of its own. Upon reaching adulthood, a male was introduced to each female’s cage and the insects were allowed to mate freely. Eggs were collected and counted every other day. After 2 weeks, the females were preserved in 95% ethanol.

DNA of the preserved adults was extracted using the DNeasy Blood and Tissue Kit (Qiagen, Germantown, MD, United States) and diagnostic PCR was performed using the Burk16sF/Burk16sR primer set (5′-TTTTGGACAATGGGGGCAAC-3′, 5′-GCTCTTGCGTAGC AACTAAG-3′; Kikuchi et al., 2005) to verify *Burkholderia* infection status. Each 10 uL PCR reaction contained 0.4 μM forward primer, 0.4 μM reverse primer, 1.6 mM dNTPs, 0.75U Taq (New England Biolabs), 1X ThermoPol buffer (NEB M0267), and 1 uL DNA extract. The thermocycler program was: Denaturation at 95°C for 4 min, followed by 40 cycles of denaturation at 94°C for 30 s, primer annealing at 55°C for 1 min, and extension at 68°C for 2 min, with a final extension of 68°C for 6 min. PCR products were visualized with gel electrophoresis. When a PCR result disagreed with the experimental treatment (i.e., *Burkholderia* was detected in an insect assigned to the aposymbiotic treatment or *Burkholderia* was not detected in an insect assigned to the symbiotic treatment), the insect was reassigned to the diagnostic PCR result.

We did not sequence the experimental insects nor the adults that served as *Burkholderia* sources in Iterations 1 and 3. However, we did sequence 16 adults from the laboratory colony from which the source adults were derived: Fourteen were monoassociated with SV1 and two were monoassociated with SV17. SV1 is either the same strain as, or a very close relative of, the TF1N1 *Burkholderia* isolate we used to infect nymphs in Iteration 2, therefore most or all of the insects in our experiments were likely infected with the same *Burkholderia* strain.

### Statistical Analyses

To explore the dominant bacteria present in *Jalysus*, we rarefied the full 16S dataset to 1000 reads per sample to control for differences among samples in sequencing depth and calculated the total number of reads assigned to each sequence variant. For the rest of our analyses, we subset the raw (unrarefied) 16S data to sequence variants assigned to the genus *Burkholderia*. In order to generate simple summary statistics (e.g., numbers of strains hosted) and pie charts, we controlled for sample-wise differences in sequencing depth by rarefying all *Burkholderia* sequences to 800 reads per sample. We chose 800 reads because it struck a balance between retaining a large number of insects in the dataset (352 individuals out of 465) and retaining a high minimum number of reads per sample for a single bacterial genus. We also generated bar plots of strain composition within individual insects using the R package “phyloseq” (McMurdie and Holmes, 2013).

For our statistical analyses, we took the full set of unrarefied *Burkholderia* sequences and discarded samples below a minimum threshold of 800 *Burkholderia* reads. We removed insects from sites with fewer than 8 collected individuals and from host plants of unknown species or from which fewer than 14 individuals were collected. To test for community-level differences in the *Burkholderia* hosted by wild *Jalysus*, Bray-Curtis distances between samples were calculated from unrarefied, proportion-normalized data. (Proportion normalization performs best for this type of test; McMurdie and Holmes, 2014.) We used PERMANOVA (the *adonis* test in the R package “vegan”; Oksanen et al., 2015) to test for dissimilarity between the insect species (*wickhami* versus *spinosus*) while accounting for differences between eastern and western regions of the United States. The same method was also used to test for differences in the *Burkholderia* community of insects on different plant species while accounting for differences between collection sites. Finally, we tested for differential abundance of individual SVs between sample groups using the R package “DESeq2” adapted for microbiome data (Anders and Huber, 2010; McMurdie and Holmes, 2014).

We used a linear model to assess how development time from hatching to adulthood was correlated with symbiont infection status (as determined by diagnostic PCR), experimental iteration, and the sex of the nymph. We used a generalized linear model with Poisson error distribution to assess how reproductive output (number of eggs laid by a female during the first 2 weeks of adulthood) was correlated with a female’s symbiont treatment and experimental iteration. We found the best-fit fixed effects structure for both models using backward model selection with likelihood ratio tests.

## Results

After sequence processing, quality filtering, and removal of contaminant sequence variants, we obtained 3,134,451 bacterial sequences from 465 insects. To verify that the insects’ whole-body bacterial communities were dominated by known insect-associated bacteria, we first rarefied the entire 16S dataset to 1000 reads per sample. After rarefaction, a total of 341 bacterial sequence variants (SVs) were detected. Many of the most abundant SVs were *Burkholderia*, but other common genera included *Wolbachia*, *Bartonella*, and *Commensalibacter*; these genera are commonly found in association with insects (Supplementary Table S4).

Next, we subset the data to the genus *Burkholderia*. After applying a minimum cutoff of 800 *Burkholderia* reads per insect, we retained 322 individuals of *J. wickhami* from 20 sites and 30 individuals of *J. spinosus* from 2 sites. We detected a total of 61 *Burkholderia* sequence variants across these individuals.

### Phylogenetic Placement of *Burkholderia* Associated With *Jalysus* spp.

**FIGURE 2:**
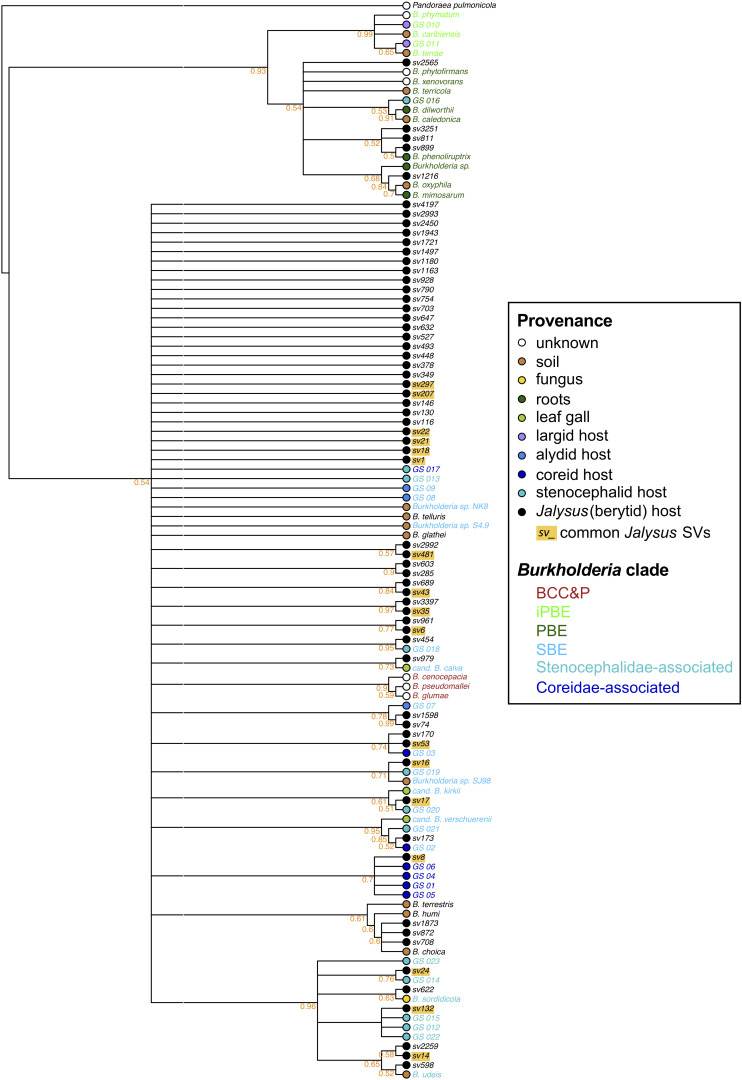
Phylogenetic placement of *Burkholderia* associated with *Jalysus* spp. Consensus maximum-likelihood phylogeny of the *Burkholderia* sequence variants detected in this study (names starting “sv” and labeled with a black dot) plus the 16S rRNA sequences of selected strains downloaded from GenBank. Where known, the source of each *Burkholderia* variant is indicated by colored dots. Text labels of GenBank strains are colored according to their membership in several named groups as proposed by Kuechler et al. (2016): SBE = stinkbug-associated beneficial and environmental; PBE = plant-associated beneficial and environmental; iPBE = the insect-associated subclade of the PBE group; BCC&P = *B. cepacia* complex and *B. pseudomallei* group; a clade associated with coreid bugs; and a clade associated with stenocephalid bugs. The most common sequence variants in *Jalysus* (those accounting for greater than 0.1% of the reads in at least five insects) are highlighted in yellow. Sequence variants from this study were 427 or 428 base pairs in length. GenBank sequences varied from 1231 to 1731 bp in length. The tree was rooted with the outgroup *Pandoraea pulmonicola*. Node support values were calculated using rapid bootstrapping which was halted automatically based on the MRE criterion. Nodes with less than 50% support have been collapsed. The corresponding best-fit maximum-likelihood tree is provided in Supplementary Figure S3.

Because Illumina amplicons are relatively short (∼427 bp) our phylogeny did not have strong support (Figure 2). However, the best-fit phylogeny (Supplementary Figure S3) recovered the same *Burkholderia* groups previously described by Kuechler et al. (2016) – specifically, the “*B. cepacia* complex and *B. pseudomallei”* (BCC&P) group; the “plant-associated beneficial and environmental” (PBE) group which contains the “insect-associated and plant-associated beneficial and environmental” (iPBE) group; and the “stinkbug-associated beneficial and environmental” (SBE) group which contains subgroups associated with the Coreidae and Stenocephalidae. However, we did not recover the same basal relationships between groups found by Kuechler et al. (2016).

According to our best-fit phylogeny, our *Burkholderia* SVs derived from all major *Burkholderia* clades except for the BCC&P, but the 17 most common sequence variants (those accounting for greater than 0.1% of the reads in at least 5 insects) were all members of the SBE clade (Supplementary Figure S3). The four most abundant sequence variants – SV1, SV6, SV8, and SV21 – appeared to be most closely related to symbionts of insects in the family Coreidae (Supplementary Figure S3).

### Alpha and Beta Diversity of *Burkholderia* Variants Associated With *Jalysus*

**FIGURE 3:**
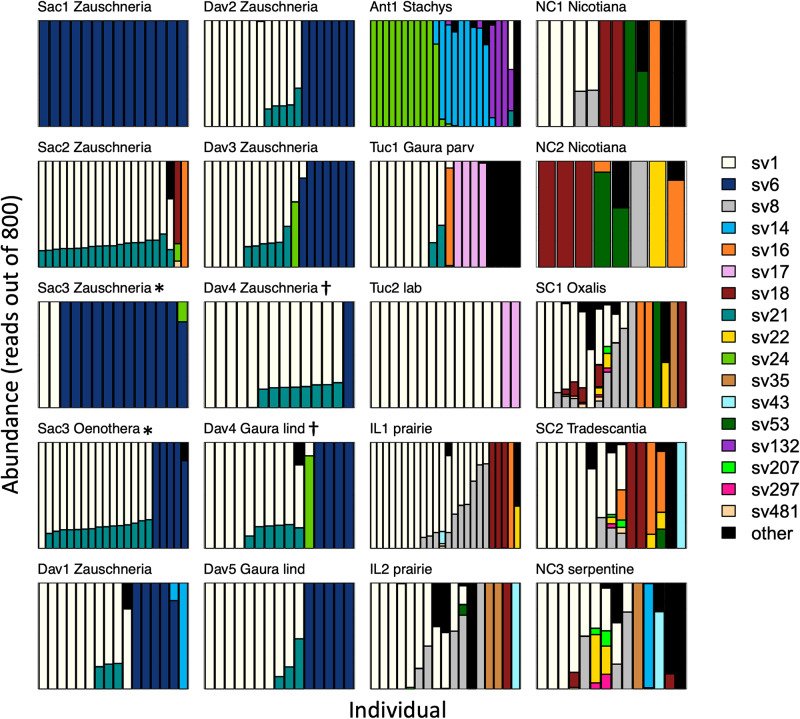
*Burkholderia* strain abundances in populations of *Jalysus* spp. Each bar represents *Burkholderia* 16S rRNA sequences derived from an individual insect after rarefaction to 800 reads, with the sequence variant indicated by color. Areas of each bar that are different colors indicate the proportion of sequences that came from different *Burkholderia* sequence variants. Rare strains (those that did not account for at least 0.1% of reads in at least five individual bugs) are colored black. Plots are titled with the collection site name plus the genus of the host plant, if known. *Gaura* species are distinguished with the first four letters of their species epithet. If the host plant was unknown (or, in the case of the laboratory population, the host was not chosen by the insects), a description of the habitat is provided instead. Plots are grouped by region, then by host plant. At two sites (Sac3* and Dav4†) insects were collected from two different host plant species. All populations from which we collected at least than eight individuals are displayed.

After rarefaction to 800 *Burkholderia* reads per sample, individual insects hosted a median of 1 (interquartile range (IQR) 1–2, max = 9) sequence variants. Sequence variant abundances were uneven within hosts, with one variant being substantially more abundant than the other(s) in each insect (Figure 3). Unexpectedly, one sequence variant, SV1, was widespread across the continental United States; it accounted for 48% of all *Burkholderia* reads. After removing sites with fewer than 8 individuals, SV1 was the most abundant sequence variant in most (13 out of 17) sites. There were only two sites in which SV1 was not detected.

**FIGURE 4:**
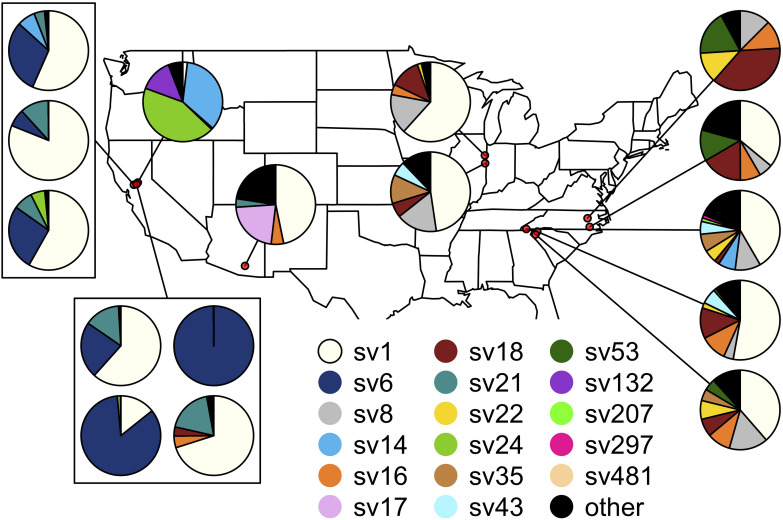
Spatial distribution of the *Burkholderia* sequence variants hosted by *Jalysus*. Each pie chart indicates population-level abundances of variants hosted by a pool of 12–24 individual bugs sampled at a single location. Rare strains (those that did not account for at least 0.1% of reads in at least five individual bugs) are colored black.

The composition of symbiont communities differed between the eastern and western United States (PERMANOVA: *df* = 1, *F* = 16.4, *p* < 0.001), though the overall amount of variation explained was small (*R*^2^ = 0.05). Seven sequence variants displayed an east-west divide in their distributions: SVs 6, 14, 21 and 24 were detected predominantly on the western side of the United States and SVs 8, 18, and 22 were largely limited to the east (DESeq2, all *p* < 0.001; Figure 4). After accounting for differences between the east and west, symbiont communities did not differ between *J. spinosus* and *J. wickhami* (PERMANOVA: *df* = 1, *F* = 0.7, *p* = 0.6).

Differences between sites accounted for a high percentage of variation in *Burkholderia* communities (PERMANOVA: *df* = 14, *F* = 7.4, *p* < 0.001, *R*^2^ = 0.27). On average, *Jalysus* within the same site were more likely to host the same sequence variant than *Jalysus* from different sites (average Bray-Curtis within populations = 0.53; average Bray-Curtis between populations = 0.74; *t* = 3.8, *df* = 27, *p* < 0.001. For reference, a Bray-Curtis dissimilarity of 1 indicates that no SVs were shared; a Bray-Curtis dissimilarity of 0 indicates that all SVs were shared at identical relative abundances).

After accounting for site differences, host plant species were also found to differ in symbiont community composition, though the variation explained was small (PERMANOVA: *df* = 2, *F* = 7.2, *p* < 0.001, *R*^2^ = 0.04). Insects collected from the same host plant species were more likely to host the same sequence variant than insects collected from different host plant species (average Bray-Curtis within plant species = 0.65; average Bray-Curtis between plant species = 0.80; *t* = 2.3, *df* = 15, *p* = 0.03). At two sites, we were able to collect insects from different host plant species in close proximity. At site Sac3, *J. wickhami* were collected from a small cluster of contiguous *Zauschneria californica* plants and from an individual *Oenothera* plant less than 5 meters away. *Burkholderia* strain SV21 was significantly associated with *Oenothera* at this site (DESeq2, *p* < 0.001; Figure 3). At site Dav4, we collected *J. wickhami* from a large patch of about 30 contiguous *Gaura lindheimeri* and from a small patch of about 5 contiguous *Zauschneria californica* plants roughly 200 meters away. No *Burkholderia* strains differed in abundance between the two host plant species at this site (DESeq2, all *p* > 0.7; Figure 3).

### *Burkholderia* Colonization Rates

We performed diagnostic PCR to check the *Burkholderia* colonization status of all insects that reached adulthood across the three iterations of the rearing experiment. In the first iteration, only four out of 14 individuals (29%) acquired *Burkholderia* in the symbiont treatment (Table 2). However, in the second and third iterations, all but one individual successfully acquired *Burkholderia* when exposed to a source of the symbiont (Table 2). Almost all insects in the aposymbiotic treatments were confirmed to be *Burkholderia*-free, however, two individuals in Iteration 3 did acquire *Burkholderia* despite lack of intentional exposure to a symbiont source (Table 2). In total, only 2/26 (8%) of nymphs that were not intentionally exposed to *Burkholderia* acquired the symbiont, while 20/32 (63%) of nymphs exposed either indirectly via adults, or directly via cultured cells acquired the symbiont (*X*^2^ = 16, *df* = 1, *p* < 0.001). For the remainder of our analyses, symbiont colonization status was assigned based on an insect’s diagnostic PCR result rather than its original experimental treatment.

**TABLE 2:** Sample sizes of nymphs that reached adulthood grouped by assigned *Burkholderia* treatment and diagnostic PCR results.

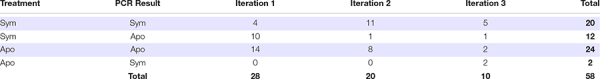

*Apo, aposymbiotic; Sym, symbiotic. Rows highlighted in blue indicate nymphs for which the diagnostic PCR result matched the assigned treatment group.*

### Effects of *Burkholderia* on Development and Fitness of *Jalysus*

**FIGURE 5:**
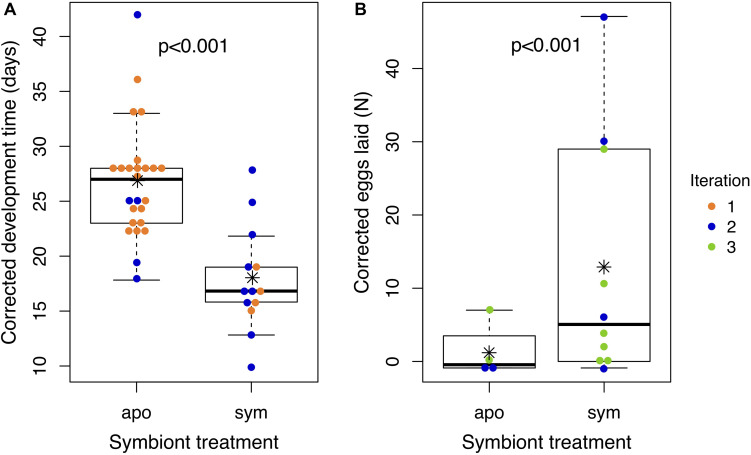
Aposymbiotic insects develop slower and lay fewer eggs than symbiotic insects. Points represent **(A)** the time an individual nymph took to develop from hatching to adulthood or **(B)** the number of eggs laid by an individual female over the first 2 weeks of adulthood. All values have been corrected for experimental iteration: development times represent model estimates if all nymphs had been reared in Iteration 1; egg numbers represent model estimates if all females had been reared in Iteration 3. Asterisks denote group means. Box plots depict medians and interquartile ranges of the data. Whiskers are placed at 1.5 times the interquartile range or, if all data fall within this range, they are placed at most extreme value measured. Corresponding raw data are displayed in Supplementary Figure S5.

We compared development time and reproductive output of *J. wickhami* reared with or without the *Burkholderia* symbiont. Due to differences in diet and rearing temperature, insects in the first iteration of this experiment developed 12.1 days faster than insects in the second iteration (*df* = 1, *t* = 6.15, *p* < 0.001). After accounting for these differences by including the experimental iteration as a factor in the model, we found aposymbiotic nymphs took 8.8 days longer to reach adulthood than symbiotic nymphs on average (*df* = 1, *t* = 4.4, *p* < 0.001; Figure 5A and Supplementary Figure S4A), representing a 34% increase in the duration of development compared to symbiotic nymphs.

Also due to differences in diet and rearing temperature, insects in the second iteration of this experiment laid 0.9 fewer eggs (*df* = 1, *z* = 5.1, *p* < 0.001) than insects in the third iteration. After accounting for these differences, aposymbiotic females laid 2.1 fewer eggs in their first 2 weeks of adulthood than symbiotic females (*df* = 1, *z* = 5.4, *p* < 0.001; Figure 5B and Supplementary Figure S4B). We also observed that aposymbiotic adults were paler and their exoskeletons appeared to be weaker and more pliable than those of symbiotic adults (Supplementary Figure S5). Pale coloration of aposymbiotic individuals has been reported in several stinkbug species (Hosokawa et al., 2006, 2013; Kikuchi et al., 2012b, 2016).

When we assigned symbiont colonization status based on the original experimental treatment rather than PCR, results for both development time and reproductive output were qualitatively identical, though differences between symbiotic and aposymbiotic insects were less pronounced (Supplementary Table S5).

## Discussion

We characterized symbiotic *Burkholderia* associated with a widespread, omnivorous stilt bug genus (*Jalysus*: Berytidae) across the United States and quantified the net fitness effects of the most common symbiont strain on *J. wickhami*. Despite high variation in the identity of the strains with which *Jalysus* associates, *Burkholderia* appears critical to the insect’s development and reproductive success. Without their symbiont, *J. wickhami* grow slower and lay fewer eggs. Aposymbiotic adults were generally paler and slower moving, suggesting potential deficits in normal melanization. Evidence that *J. wickhami* depends on a symbiont that is putatively nutritional is especially intriguing given that these stilt bugs are omnivorous and we provided the insect with unlimited protein in our experiments. In this respect, this system is very different from most nutritional symbioses that serve to complement an indigestible, unbalanced or nitrogen poor diet, for example of plant sap, wood, or blood (Buchner, 1965). Even though *J. wickhami*’s diet is nitrogen rich, the insect still depends on *Burkholderia*. Given the evidence that *Burkholderia* in gut crypts can recycle nitrogenous wastes such as allantoin and urea, as well as produce essential amino acids and B vitamins (Ohbayashi et al., 2019) stilt bugs might assimilate nutrients more efficiently with their *Burkholderia* symbiont, even in the presence of ample protein. Perhaps, as in a weevil-*Nardonella* symbiosis, the symbiont boosts the supply of a particular nutrient beyond levels that feeding or host synthesis could provide during critical developmental timepoints (Anbutsu et al., 2017). It is also possible that *Burkholderia* synthesizes a non-nitrogenous nutrient for *Jalysus*, and recent results suggest that hosts may receive many different nutrients simultaneously via digestion of *Burkholderia* cells in the midgut region directly anterior to the M4, the M4B (Ohbayashi et al., 2019). Alternatively, it’s possible that the long association between stilt bugs and *Burkholderia* has resulted in relaxed selection on the host insect’s genome for synthesis of a critical nutrient that *Burkholderia* provides, regardless of the abundance of the nutritional building blocks in the bug diet.

As has been found in other bug-*Burkholderia* symbioses, the relationship between *Jalysus* and *Burkholderia* appeared highly promiscuous. *Jalysus* associated with over 60 symbiont strains and these did not differ consistently between the sister insect species (*wickhami* and *spinosus*). Although one strain, SV1, was dominant and geographically widespread across insect populations, others were more localized: seven sequence variants were associated with either eastern or western sites. However, differences between the east and west explained a small amount (5%) of the total variation in symbiont communities. Insects that fed on different host plant species did tend to host different *Burkholderia*, but this also only accounted for a small amount (4%) of the variation in hosted symbiont communities. Furthermore, at two sites where we sampled multiple plant species, only one or zero strains, respectively, differed in abundance between the plant species. In contrast, the specific location at which an insect was collected explained a large amount (27%) of the variation in symbiont strains. Together, these results suggest that *Burkholderia* does not play an important role in these insects’ host plant specificity, but local factors drive which strains *Jalysus* acquires at a fine spatial scale.

This raises the question of how *Jalysus* acquires *Burkholderia.* The bean bug, *Riptortus pedestris*, acquires *Burkholderia* directly from the soil (Kikuchi et al., 2007). Fine-scale variation in the *Burkholderia* strains associated with *Jalysus* may therefore result from highly localized changes in strain abundances in the environment, for example from soil underneath the plant, and/or dust with bacterial cells blowing onto the plant. Another local source could include the tissue of the host plant itself. It is possible that plants could become infected with a particular *Burkholderia* strain (or strains); insects that colonize a plant could then be more likely to acquire the host plant’s strain (Kaltenpoth and Flórez, 2020).

Alternatively, nymphs might acquire the symbiont vertically (from their parents) or horizontally from conspecific adults, at least some of the time. In our rearing experiments, 2 out of 26 nymphs acquired *Burkholderia* without intentional exposure to a symbiont source. It is possible that they acquired *Burkholderia* by vertical transmission via the egg surface, as occurs in symbioses between *Burkholderia* and blissid bugs (Boucias et al., 2012; Itoh et al., 2014) and perhaps also largid bugs (Takeshita et al., 2015). However, in our early work with *J. wickhami* we performed diagnostic PCR on a small number of eggs and never detected *Burkholderia*. Furthermore, the two unexpectedly *Burkholderia*-positive nymphs were reared together in the same box for their first four instars. They may therefore have acquired *Burkholderia* via contamination of the rearing box rather than vertical transmission.

Although evidence for occasional vertical transmission via the egg is equivocal, we found that horizontal bug-to-bug transmission can occur in *J. wickhami*, at least in the lab. In our rearing experiments, roughly one third of nymphs acquired *Burkholderia* when directly exposed to a single adult male and over 80% of nymphs acquired *Burkholderia* when reared on leaves that had previously hosted six adult insects. This demonstrates that adult-to-nymph transmission is not only possible, but likely at high insect densities and in the absence of another symbiont source. This is surprising given that bug-to-bug transmission was never observed in the bean bug, *R. pedestris* (Alydidae) despite continuous exposure of 107 nymphs to adults (Kikuchi et al., 2007) and suggests that the ability to transmit *Burkholderia* varies among the hemipteran families.

*Jalysus*’ transmission capacity might be sex-specific. In Iteration 1 we used adult males as the *Burkholderia* source, whereas in Iteration 3 we used groups of six “source” adults that included males and females. Furthermore, source females were allowed to lay eggs (which were removed prior to introduction of the experimental nymphs). While the high *Burkholderia* colonization rate in Iteration 3 was likely due to the higher density of source adults, it is also possible that adult females transmit *Burkholderia* more successfully that adult males, and they might transmit it most actively while they are laying eggs. While highly speculative, either or both phenomena could be evolutionarily favorable given the demonstrated benefits *Burkholderia* provides to *J. wickhami*.

Overall, our results indicate that in the lab, (1) direct vertical transmission of *Burkholderia* is uncommon, if it occurs at all; (2) adult *J. wickhami* are capable of horizontal symbiont transmission; (3) the probability of transmission increases with the density of *Burkholderia*-colonized adults; and (4) transmission can occur without direct contact between nymphs and adults. This suggests that horizontal transmission of *Burkholderia* can occur via enrichment of the symbiont in the local environment by adult insects. While this possibility has been proposed previously (Itoh et al., 2018) to our knowledge this is the first empirical evidence that it can occur in a bug-*Burkholderia* symbiosis. It seems plausible that horizontal transmission via environmental enrichment also happens in the wild, given the insects’ tendency to aggregate. Although *Jalysus* are not social, they often feed together in groups, sometimes clustering in multi-generation aggregations on flower stalks (Figure 1). Older individuals colonized by *Burkholderia* could therefore indirectly transmit the symbiont to nymphs by several mechanisms. They could inoculate water droplets on the plant, the leaf surface, or the internal tissues of the plant via feeding or feces (although related insects do not excrete live *Burkholderia* in their feces; Kikuchi et al., 2007) or they could inoculate the soil underneath the plant when they die, fall to the ground and decompose (though this mechanism was less likely in our lab since we removed dead insects from the cages). Occasional adult-to-nymph transmission could contribute to several of the patterns we observed, including the high similarity in strains hosted by insects collected at the same location and the prevalence of SV1 across most insect populations. Further research on the relative importance of these modes of *Burkholderia* acquisition will help elucidate the ecological maintenance and potential evolutionary origin of bug-*Burkholderia* symbioses.

Finally, many of the insects in our study appeared to host two, three, or even more strains of *Burkholderia*. The fact that we extracted DNA from whole insect bodies might contribute to this pattern: although *Burkholderia* are usually housed in a specific location in the gut (crypts in the M4 region), some of the sequence variants observed in this study could have been located elsewhere in the insects’ bodies. In a few cases, the appearance of multiple strains could be an artifact of the DADA2 algorithm. While powerful, any algorithm that differentiates at the level of a single base pair may occasionally mistake a strain with a single nucleotide polymorphism for two different strains. If this were the case, we would expect to see a consistent pair of strains present at the same relative abundances across insects. We do see this pattern with SV1 and SV21 (Figure 3). This pattern of co-occurrence is intriguing but therefore must be treated cautiously and requires further experimental investigation. However, many of the strains observed in multiply-infected insects did not display consistent pairings and frequencies, suggesting in these instances at least, multiple strains were indeed present. Multiple *Burkholderia* symbiont strains have been cultured from single insects in other bug-*Burkholderia* symbioses (Kikuchi et al., 2011) and, in general, multiple strain infection is more frequent in symbioses that involve horizontal or environmental acquisition (Ebert, 2013). Within the M4 different strains might occupy different crypts or compartments, facilitating coexistence. The presence of multiple symbiont strains within some individuals raises additional questions about the factors that determine which strain (or strains) an insect will host. *Burkholderia* strains belonging to the SBE (stinkbug-associated beneficial and environmental) clade have been shown to outcompete other clades of *Burkholderia* when colonizing the stinkbug gut (Itoh et al., 2019). Do SBE-clade *Burkholderia* strains also compete with one another? Is multiple-strain colonization – and, potentially, competition – helpful or harmful to the host? Can strains displace each other? Do priority effects affect which strain colonizes an insect? These are fruitful avenues for future research.

We cannot yet say what associating with a variety of *Burkholderia* strains means functionally for the host. *Jalysus* may associate with many strains of *Burkholderia* because all strains are functionally equivalent to the insect. If this is the case, abundances of strains in an insect population might reflect abundances of SBE-clade *Burkholderia* in their local environment. However, strains likely vary in their competitive ability to colonize the host, as has been previously shown in *R. pedestris* (Itoh et al., 2019) which could skew strain abundances relative to the environmental pool even if strains are functionally equivalent from the insect’s perspective. Alternatively or additionally, the benefits provided by different strains may be context-dependent. Perhaps strains that are highly abundant in western populations are more beneficial in western climates, eastern strains are, similarly, particularly beneficial in eastern climates, while SV1 (the widespread strain) is moderately beneficial across all climates. It is uncertain whether *Jalysus* can actively select strains matched to local conditions, however, a passive process could still result in local adaptation. For example, if insects acquire strains based on their frequency in the environment but some strains are more beneficial, the individuals that happen to host the “better” strains are more likely to survive and will therefore dominate the insect population. Furthermore, our rearing experiments suggest that adult *Jalysus* can transmit the symbiont to nymphs via enrichment of the local environment, and therefore may provide a baseline reservoir of *Burkholderia* for nymphs in their immediate vicinity.

## Data Availability Statement

Raw Illumina sequences are available on the NCBI Sequence Read Archive under BioProject number PRJNA613319. All other data (*Jalysus* rearing data, Illumina SV table, SV taxonomic assignments, representative sequences, and sample metadata) are available as Supplementary Material.

## Author Contributions

AR and MH designed the study. AR and MT collected the samples. AR performed the experiment, processed the samples, analyzed the data, and wrote the first draft of the manuscript. All authors contributed to manuscript revision and read and approved the submitted version.

## Conflict of Interest

The authors declare that the research was conducted in the absence of any commercial or financial relationships that could be construed as a potential conflict of interest.
