# Supplementary material for: Continent-Scale Sampling Reveals Fine-Scale Turnover in a Beneficial Bug Symbiont
Source: Front Microbiol. 2020 Jun 19;11:1276. doi: 10.3389/fmicb.2020.01276 (PMC7316890; doi:10.3389/fmicb.2020.01276)
Supplement: Supplementary file 1 [file Data_Sheet_1.zip › READ ME.rtf]

Paper: Continent-scale sampling reveals fine-scale turnover in a beneficial bug symbiontJournal: Frontiers in MicrobiologyAuthors:  Alison Ravenscraft, Margaret W. Thairu, Alison K. Hansen, Martha S. HunterDescription: This README file describes the data package accompanying the above publication.Files in Jalysus2020_Data:1. Jalysus2020_repset.fasta: Representative FASTA sequences for all of the 16S rRNA amplicon sequence variants detected in this study (includes Burkholderia and all other bacterial genera). These representative sequences were determined by the DADA2 algorithm.2. Jalysus2020_taxtable.csv: Taxonomic identifications of the sequence variants. Taxonomy was assigned by the RDP classifier using the SILVA nr v123 database as thetraining set. The ‘seqlen’ column reports the length of each SV’s representative sequence in base pairs.3. Jalysus2020_SVtable.csv: The count (number of reads) of each sequence variant in each sample prior to rarefaction. Columns are samples, rows are bacterial SVs. Samples are named according to the barcodes that were assigned to them during the Illumina runs. Chloroplasts and mitochondria have been removed, sequences less than 401 base pairs in length have been removed, and contaminant SVs (identified by the R “decontam” package) have been removed.4. Jalysus2020_metadata.csv: Sample metadata for insects that were sequenced on the Illumina runs. Column descriptions:barcode: The forward and reverse barcode combinations that each sample was assigned during the Illumina runs.sampleid: The identifying number assigned to each sample as it was collected.bugspecies: The insect species.bug.family: Taxonomic family of the insect.hostplant: Plant species on which the insect was collected.instar: Developmental stage of the insect. Nymphal instars are numbered in increasing order.sex: Sex of the insect, if adult.inst.sex: Same as the instar column except that the sex of adults is specified.site: Name of the site where the insect was collected. Most site names match those in Supplementary Table S1. However, sites from which fewer than 8 individuals were collected have been given names that do not appear in Table S1; these were sites at which <8 insects were collected, therefore they were not included in the statistical analyses. Furthermore, insects were not included in the statistical analyses or figures if they were collected off a “rare” host plant.  (For example, one individual at site Ant1 was collected from Salvia; this individual is not included in the analyses or figures.)run: indicates which Illumina run the sample was sequenced on.is.blank: Indicates whether a sample was an insect (“sample”) or a PCR or extraction blank (“blank”).5. Jalysus2020_devtime.csv: Experimental rearing data - effect of Burkholderia on nymph development time. Column descriptions:bugid: Identity of the nymph.first, second, third, fourth, fifth: Duration in days of each of the instars indicated by the column titles.dev: Total duration of development in days from hatching to the first day of adulthood.sex: Sex of the nymph. Only known for nymphs that reached adulthood.assigned.trtmt: Assigned symbiont status of the nymph. Apo= aposymbiotic, Sym= symbiotic.pcr.result: Actual symbiont status of the nymph as determined by diagnostic PCR. This information isn’t available for all nymphs that died during development, but it is available for all individuals that reached adulthood adults. Only individuals that reached adulthood were included in the analyses.iteration: The experimental iteration. Rearing conditions for each iteration are described in Table 1.diet: The protein source offered to the insects.temp: Temperature at which the nymph was reared (see Table 1).6. Jalysus2020_eggs.csv: Experimental rearing data - effect of Burkholderia on adult female reproductive fitness. Column descriptions:bugid: Identity of the nymph.boxid: Identity of the box in which the nymph was reared from the 1st through 4th instars. (This is only relevant for Iteration 3 bugs, which were reared in groups during the early instars but separated into individual boxes at the fifth instar.)fitness: Number of eggs laid over the first two weeks of adulthood.assigned.trtmt: Assigned symbiont status of the nymph. Apo= aposymbiotic, Sym= symbiotic.pcr.result: Actual symbiont status of the nymph as determined by diagnostic PCR. This information isn’t available for all nymphs that died during development, but it is available for all individuals that reached adulthood adults. Only individuals that reached adulthood were included in the analyses.iteration: The experimental iteration. Rearing conditions for each iteration are described in Table 1.diet: The protein source offered to the insects.temp: Temperature at which the nymph was reared (see Table 1).
